# Latent profile analysis of health literacy among breast cancer patients and comparative analysis of quality of life disparities

**DOI:** 10.3389/fonc.2026.1769721

**Published:** 2026-04-23

**Authors:** Mengwei Jiang, Bei Wu, Yi Liu, Detian Liu, Xiaoye Chen, Ting Shu, Zijun Yuan, Linxin Xie, Hongzhen Xie

**Affiliations:** 1 Department of Health Medicine, General Hospital of Southern Theater Command, Guangzhou, Guangdong, China; 2 General Surgery, General Hospital of Southern Theater Command, Guangzhou, Guangdong, China; 3 School of Nursing, Guangdong Pharmaceutical University, Guangzhou, Guangdong, China; 4 Department of Cardiology, Nanxishan Hospital (the Second People's Hospital) of Guangxi Zhuang Autonomous Region, Guilin, Guangxi, China; 5 School of Nursing, Southern Medical University, Guangzhou, Guangdong, China; 6 School of Medicine, Yangtze University, Jingzhou, Hubei, China

**Keywords:** breast cancer, factors, health literacy, latent profile analysis, quality of life

## Abstract

**Objective:**

This study aimed to explore the latent profiles of health literacy among breast cancer patients and examine their relationship with quality of life.

**Methods:**

A total of 182 patients were consecutively recruited from the general surgery department using a convenience sampling method. Data were collected using a general information questionnaire, the Chronic Disease Health Literacy Scale, and the Breast Cancer Quality of Life Scale. Latent profile analysis (LPA) was conducted using Mplus 8.3 to identify distinct health literacy profiles. Subsequently, SPSS 27.0 was used to compare differences in quality-of-life scores the identified profiles.

**Results:**

Three distinct latent profiles of health literacy were identified among breast cancer patients: a low health literacy profile (9.2%), a moderate health literacy profile (29.3%), and a high health literacy profile (61.5%). Multivariate logistic regression analysis indicated that educational attainment, primary caregiver status, and having relatives or friends working in healthcare were significantly associated with health literacy profile membership (all *P* < 0.05). Furthermore, the overall quality-of-life score and all subscale scores differed significantly across the three profiles (all *P* < 0.05).

**Conclusion:**

This study identified three distinct health literacy profiles among breast cancer patients, which were significantly associated with different levels of quality of life. To optimize patient outcomes, clinical nursing practice should develop and implement tailored interventions based on these specific profile characteristics.

## 1 Introduction

Breast cancer is the most common cancer among women, accounting for 11.6% of all new cancer cases worldwide (1). In China, deaths from breast cancer account for 18.4% of global breast cancer mortality (2). Ongoing advances in medical paradigms, diagnostic strategies, and therapeutic modalities have raised the 5-year survival rate among breast cancer patients to 82.0% (3). Despite prolonged survival, quality of life remains at a low to moderate level due to unresolved issues related to the disease itself and chemotherapy, including problems with body image, physical health, and emotional well-being (4). Research indicates that quality of life has become a key indicator for assessing prognosis (5). Health literacy has emerged as a critical determinant of quality of life (6). Health literacy refers to an individual’s ability to obtain, understand, and process basic health information or services, and to make appropriate health-related decisions (6, 7). An Iranian study found that 89.6% of breast cancer patients had insufficient health literacy, and this issue is also prominent among Chinese breast cancer patients (8). Evidence indicates that health literacy among breast cancer patients in China remains suboptimal (9). Patients with low health literacy exhibit inadequate comprehension of medical information and poor health behaviors, leading to delayed access to timely medical care. This delays optimal treatment opportunities, increases the incidence of related complications, raises healthcare costs, and ultimately results in a diminished quality of life (4, 6, 10). Therefore, enhancing the health literacy of breast cancer patients has become a beneficial pathway to improving their quality of life.

The Knowledge-Belief-Action Theory states that individuals acquire knowledge and information, then actively process and reflect on them to form beliefs and attitudes, thereby driving behavioral change (11). In this study, the health literacy scale includes four dimensions: information acquisition ability and communication interaction ability serve as the foundation for individuals to obtain knowledge and information; willingness to provide economic support represent beliefs and attitudes. These components jointly promote subsequent health behavior changes and further improve quality of life. When patients possess sufficient disease prevention knowledge and health literacy skills, they can effectively manage their own health—which is a proven pathway to enhancing quality of life (12). Currently, research on health literacy among breast cancer patients both domestically and internationally has primarily focused on describing the current situation, exploring influencing factors, and examining mediating effects (13–15), Previous studies have not fully accounted for heterogeneity within the patient population, and the relationship between individual health literacy levels and quality of life differences has not been explored in depth. Latent profile analysis (16) is an individual-centered approach that classifies individuals based on their distinct characteristics, enabling the analysis of group heterogeneity. Therefore, this study aims to identify latent profiles of health literacy among breast cancer patients using LPA and to compare differences in quality of life across these groups. This approach seeks to provide a theoretical basis for developing targeted interventions to enhance health literacy among breast cancer patients, thereby improving their quality of life.

## 2 Objects and methods

### 2.1 Research subjects

A total of 182 breast cancer patients were enrolled using convenience sampling from Grade III Class A hospitals in Guangdong Province and Guangxi Zhuang Autonomous Region between October 2024 and February 2025. The inclusion criteria were as follows: ① female patients aged ≥18 years; ② diagnosis of breast cancer according to the Guidelines and Standards for Diagnosis and Treatment of Breast Cancer (2024 Edition) issued by the Chinese Anti-Cancer Association (17); ③ provision of written informed consent and voluntary participation in this study. The exclusion criteria were as follows: ① individuals with language, hearing, cognitive, or intellectual impairments, or those in poor physical condition and unable to cooperate with the investigation. According to Kendall’s sample size calculation method, the sample size should be at least 5 to 10 times the number of variables (18), with an additional 20% to account for potential attrition. This study included a total of 21 independent variables, yielding a required sample size range of 126–252. A final sample of 182 participants was included. Previous studies have successfully conducted latent profile analysis using 14 indicators with 139 participants (19). In the present study, latent profile analysis was based on 4 indicators and 182 participants, suggesting that the sample size was sufficient to identify distinct subgroups. This study was approved by the hospital ethics committee [NZLLKZ2024086].

### 2.2 Methods

#### 2.2.1 Survey methodology

Prior to the study, investigators responsible for questionnaire administration completed standardized training. The trained research staff then conducted the survey using paper-based questionnaires. After obtaining consent from hospital administrators, the research team entered the relevant departments to distribute the questionnaires. They used a standardized script to explain the study purpose, procedures, and significance to participants. All questionnaires were completed and collected on-site. A total of 190 questionnaires were distributed, and 182 valid responses were collected, yielding a valid response rate of 95.8%.

#### 2.2.2 Survey questionnaire

The questionnaire was developed based on a literature review and expert panel discussions: (1) General Information Questionnaire: This section included: ① Basic Information: Age, educational background, primary residence, primary caregiver, marital status, employment status, financial status; ② Medical History: Disease progression, disease staging, family history of hereditary conditions, surgical history, chemotherapy history, wound healing status, and long-term indwelling intravenous catheter status; ③ Social Support: Whether relatives or friends work in healthcare, and participation in breast cancer support groups. (2) Health Literacy Measure for Chronic Disease Patients (HeLMS): This scale was developed by Sun Haolin et al. (20) and adapted from the Health Literacy Measurement Scale (HeLMS) developed by Jordan et al. Although initially designed for the general chronic disease population, this scale has been validated and widely used among Chinese breast cancer survivors (21). This scale consists of 24 items covering information acquisition ability (9 items), communication and interaction ability (9 items), willingness to improve health (4 items), and willingness to provide financial support (2 items). Each item is rated on a 5-point Likert scale, with responses scored from 1 (“extremely difficult”) to 5 (“not difficult at all”) and total scores ranging from 24 to 120. The Cronbach’s α coefficient for this scale in the present study was 0.929. (3) Functional Assessment of Cancer Therapy–Breast (FACT-B): This scale was translated and revised by Chonghua Wan et al. (22) based on the Functional Assessment of Cancer Therapy system developed by Professor Celia. The scale comprises five dimensions and 36 items: - Physical Condition (7 items) - Social and Family Situation (7 items) - Emotional State (6 items) - Functional Status (7 items) - Additional Concerns (9 items). All items are rated on a five-point Likert scale ranging from “Not at all” to “Very much, “with scores ranging from 0 to 4 and total scores ranging from 0 to 144. The Cronbach’s α coefficient for this scale in the present study was 0.899.

### 2.3 Statistical methods

This study utilized Excel 2010 for data screening and cleaning, and SPSS 27.0 for statistical analysis. Normally distributed continuous data were presented as mean ± standard deviations. The t-test was used for comparisons between two groups, and analysis of variance (ANOVA) was used for comparisons among three or more groups. Non-normally distributed continuous data were described using medians (P25, P75) and compared using the Kruskal–Wallis H test. Categorical data were presented as frequencies and percentages, and were analyzed using the chi-square test or Fisher’s exact test. Unordered multinomial logistic regression analysis was performed to identify factors associated with health literacy profile membership. A two-sided significance level of α = 0.05 was considered statistically significant.

Latent profile analysis (LPA) of health literacy profiles among breast cancer patients was performed using Mplus 8.3 software. Key model fit indices included the following: ① Akaike Information Criterion (AIC), Bayesian Information Criterion (BIC), and sample-size adjusted BIC (aBIC): lower values indicate a better-fitting model; ②Entropy: values closer to 1 indicate higher classification accuracy; ③ Likelihood ratio test (LMR) and bootstrap likelihood ratio test (BLRT): these tests were used for model comparison, with *P* < 0.05 indicating that the k-class model fits significantly better than the (k–1)-class model.

## 3 Result

### 3.1 General characteristics of breast cancer patients

Among 182 breast cancer patients, ages ranged from 18 to 85 years (mean ± standard deviation: 51.81 ± 10.54 years). Regarding educational attainment: 62 patients with less than elementary school education, 61 patients with junior high school, senior high school, or technical secondary school education, and 59 patients with college or undergraduate education; regarding residence, there were 94 urban patients and 88 rural patients; regarding marital status, there were 174 married patients, 6 unmarried patients, and 2 patients with other marital statuses; regarding employment status, there were 132 employed patients and 50 unemployed patients; regarding disease duration, 155 patients had a duration of <24 months, and 27 had a duration of ≥24 months; regarding disease stage, 51 patients were at stage 0–I, 66 at stage II, and 65 at stage III–IV. Additionally, 41 patients had comorbid chronic diseases, 130 had a history of surgery, and 133 had a history of chemotherapy.

### 3.2 A potential profile analysis of health literacy among breast cancer patients

A latent profile model with 1 to 4 classes was constructed based on the four dimensions of the Health Literacy Scale: information acquisition ability, communication and interaction ability, willingness to improve health, and willingness to provide financial support. As the number of classes increased, AIC, BIC, and aBIC values gradually decreased. Both the LMR and BLRT were statistically significant (*P* < 0.05), with entropy values of 0.867, all exceeding 0.8. Although Model 4 exhibited the smallest information fit index values, its LMR P-value of 0.323 indicated a poor model fit. Model 2 exhibited higher fit index values than Model 4, with an Entropy value of 0.864, yet its classification accuracy was lower than that of Model 3. When the number of classes was 3, the Entropy value reached 0.873, and both the LMR and BLRT were statistically significant. After comprehensive consideration, Model 3 was selected as the optimal profile model, as shown in **Table 1**.

**Table 1:** Model fit measures for latent profiles of health literacy among breast cancer patients.

Model	AIC	BIC	aBIC	Entropy	*P*		Category probability(%)
					LMR	BLRT	
1	1684.456	1710.088	1684.751	-	-	-	
2	1491.557	1533.209	1492.037	0.864	0.012	<0.001	71.82/28.18
3	1407.779	1465.451	1408.443	0.873	0.002	<0.001	61.55/29.28/9.17
4	1366.923	1440.615	1367.771	0.912	0.323	<0.001	57.81/13.58/20.22/8.4

### 3.3 Naming of latent profiles of health literacy among breast cancer patients

**Figure 1:**
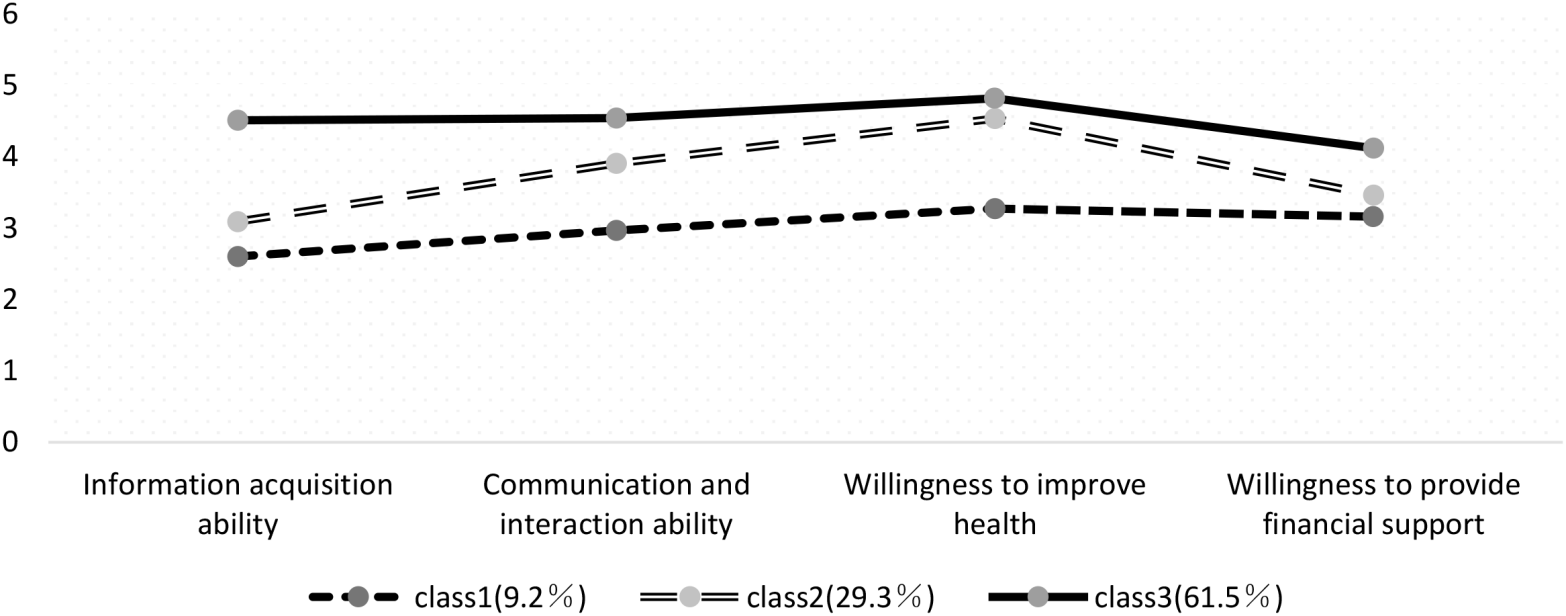
Distribution of characteristics across three latent profiles of health literacy among breast cancer patients.

As shown in **Figure 1**, each category was named according to its characteristics: Category 1 (C1) had the lowest score on the information acquisition ability dimension and showed a significant gap compared to the other two categories. It was designated as the low health literacy group, with a score of 2.88 (2.64, 3.11), accounting for 9.2% of the study participants. Category 2 (C2) scored between C1 and C3 across all four dimensions. Based on its scoring characteristics, it was designated as the moderate health literacy group, with a score of 3.65 (3.42, 3.88), accounting for 29.3% of the study population. Category 3 (C3) had the highest overall scores across all dimensions and was therefore designated as the high health literacy group, with a score of 4.58 (4.33, 4.76). This category accounted for 61.5% of the study participants.

### 3.4 Comparison of quality of life among breast cancer patients across different health literacy profiles

The comparison of quality-of-life scores among breast cancer patients across the three health literacy profiles is presented in **Table 2**. *Post-hoc* Bonferroni tests revealed statistically significant differences in overall quality of life scores (*P* < 0.05) between the low health literacy group (C1) and the moderate health literacy group (C2), and between the low health literacy group (C1) and the high health literacy group (C3).

**Table 2:** Comparison of quality-of-life scores among breast cancer patients across different health literacy profiles, M(P_25_, P_75_).

Dimension	Cross-section			*Hc*	*P*
	C1	C2	C3		
Physiological Condition Dimension	2.43(1.61, 3.11)	2.93(2.18, 3.54)	3.00(2.57, 3.46)	6.295	0.043
Social and Family Dimension	1.93(1.57, 2.79)	3.07(2.71, 3.29)	3.00(2.11, 3.43)	9.466	0.009
Emotional Status Dimension	1.83(1.50, 3.46)	2.50(1.88, 3.17)	2.83(2.17, 3.33)	6.619	0.037
Functional Status Dimension	1.71(1.43, 2.14)	2.64(2.04, 3.00)	2.86(2.00, 3.43)	10.624	0.005
Additional Focus Dimensions	2.33(1.89, 2.75)	2.44(2.22, 2.78)	2.67(2.33, 3.11)	8.434	0.015
Overall Quality of Life Score	2.13(1.94, 2.44)	2.72(2.28, 2.97)	2.86(2.33, 3.20)	13.176	0.001

C1, Low health literacy; C2, Moderate health literacy; C3, High health literacy.

### 3.5 Univariate analysis of health literacy profiles among breast cancer patients

Comparisons among patients across different health literacy profiles revealed statistically significant differences in educational attainment, primary residence, primary caregiver, economic status, and whether relatives or friends worked in healthcare (all *P* < 0.05). Detailed results are presented in **Table 3**.

**Table 3:** Comparison of health literacy profiles by demographic variables(n=182).

Project	Category	C1(n=16)%	C2(n=52)%	C3(n=114)%	*X^2^*	*P*
Age (years)	<45	2(12.5)	10(19.2)	27(23.7)	4.708	0.319
	45~60	13(81.3)	29(55.8)	65(57.0)		
	≥61	1(6.2)	13(25.0)	22(19.3)		
Educational attainment	Below elementary school	12(75.0)	17(32.7)	33(28.9)	17.229	0.002
	Junior High School - Vocational High School	2(12.5)	13(25.0)	46(40.4)		
	Associate’s/Bachelor’s degree	2(12.5)	22(42.3)	35(30.7)		
Permanent residence	Town	4(25.0)	24(46.2)	66(57.9)	6.960	0.031
	Rural	12(75.0)	28(53.2)	48(42.1)		
Primary caregiver	Spouse	4(25.0)	32(61.5)	71(62.3)	8.276	0.016
	Non-spouse	12(75.0)	20(38.5)	43(37.7)		
Marital Status	Unmarried	0(0.0)	0(0.0)	6(5.3)	4.229	0.376
	Married	16(100.0)	51(98.1)	107(93.9)		
	Others	0(0.0)	1(0.2)	1(0.8)		
Current employment status	Yes	15(93.8)	33(63.5)	84(73.7)	5.838	0.054
	No	1(6.2)	19(36.5)	30(26.3)		
Economic conditions	≤5000	9(56.3)	35(67.3)	45(39.5)	11.451	0.003
	>5000	7(43.7)	17(32.7)	69(60.5)		
chronic disease	Yes	4(25.0)	13(25.0)	24(21.1)	0.380	0.827
	No	12(75.0)	39(75.0)	90(78.9)		
Course of the disease	<24 months	12(75.0)	47(90.4)	96(84.2)	2.512	0.285
	≥24 months	4(25.0)	5(9.6)	18(15.8)		
Disease Staging	Stage 0-I	4(25.0)	10(19.2)	37(32.4)	5.512	0.239
	Phase II	5(31.3)	25(48.1)	36(31.6)		
	Stage III-IV	7(43.7)	17(32.7)	41(36.0)		
Surgical Procedure	Yes	11(68.7)	41(78.8)	78(68.4)	1.963	0.375
	No	5(31.3)	11(21.2)	36(31.6)		
Chemotherapy	Yes	12(75.0)	41(78.8)	80(70.2)	1.398	0.497
	No	4(25.0)	11(21.2)	34(29.8)		
Wound condition	No wounds	4(25.0)	11(21.2)	22(19.3)	5.609	0.468
	Partial healing	7(43.7)	22(42.3)	33(28.9)		
	Fully healed	5(31.3)	17(32.7)	53(46.5)		
	Unhealed	0(0.0)	2(3.8)	6(5.3)		
Long-term intravenous access device	Yes	5(31.2)	19(36.5)	41(36.0)	0.157	0.924
	No	11(68.8)	33(63.5)	73(64.0)		
Friends and relatives working in healthcare	Yes	5(31.3)	33(63.5)	77(67.5)	7.948	0.019
	No	11(68.8)	19(36.5)	37(32.5)		
Attended a patient support group	Yes	2(12.5)	8(15.4)	24(21.1)	1.196	0.550
	No	14(87.5)	44(84.6)	90(78.9)		

### 3.6 Multivariable analysis of potential categories of health literacy among breast cancer patients

Using health literacy profiles among breast cancer patients as the dependent variable, logistic regression analysis was conducted with statistically significant indicators from univariate analysis (educational attainment, place of residence, primary caregiver, economic status, and whether friends or relatives work in healthcare) as independent variables. The dependent variable was coded as follows: low health literacy group (C1) = 1, moderate health literacy group (C2) = 2, and high health literacy group (C3) = 3. The independent variables were coded as follows: elementary school or below = 0, Junior high, high school, or vocational school = 1, College or university = 2; Primary caregiver: Non-spouse = 0, Spouse = 1; Whether relatives or friends work in healthcare: No = 0, Yes = 1. All independent variables were entered into the model as categorical variables. The results are shown in **Table 4**.

**Table 4:** Multivariable logistic regression analysis of health literacy profiles among breast cancer patients(n=182).

Independent variable	Category			C2 vs. C1				C3 vs. C1		
			*B*	OR	95%CI	*P*	*B*	OR	95%CI	*P*
Educational attainment		Elementary school and below	-2.072	0.126	0.023-0.689	0.017	-1.752	0.173	0.033-0.919	0.039
Primary caregiver		Non-spouse	-1.483	0.227	0.059-0.880	0.032	-1.456	0.233	0.063-0.867	0.030
Friends and relatives working in the medical field		No	-1.430	0.239	0.066~0.866	0.029	-2.244	0.106	0.014~0.831	0.033

## 4 Discussion

### 4.1 Latent profile characteristics of health literacy among breast cancer patients

Through latent profile analysis, it was found that the overall health literacy of breast cancer patients could be categorized into three latent profiles, with significant individual variability observed. The low health literacy group accounted for 9.2%. Patients in this profile scored had lower scores than the other two groups across all dimensions of health literacy, with the lowest scores observed in the information acquisition ability dimension. Specifically, these patients showed deficiencies in their ability to filter useful information and understand health-related information, which may be related to educational attainment and place of residence (15). Within this latent profile, 75.0% were from rural areas, and 68.8% had no family members or friends engaged in the medical field. Research indicates that patients residing in rural areas tend to have lower levels of health literacy, with only 13.3% demonstrating awareness of breast cancer prevention and treatment knowledge (23). On the one hand, this may be due to limited and relatively narrow channels for acquiring disease-related knowledge. Studies focusing on Chinese breast cancer patients show that 82.7% of patients’ health information comes from social media, while only 25.9% is obtained from hospitals. This pattern further highlights inadequate reliance on formal medical information channels. Notably, 84.2% of the patients in this study were urban residents, who generally have higher internet penetration and more frequent social media usage (21). On the other hand, long-standing reliance on passive medical interactions for obtaining health information, coupled with underutilization of proactive preventive information channels (such as community outreach and the internet), may also contribute to this phenomenon. The present study further revealed that 87.5% of patients in this low health literacy group had never participated in any patient support group, indicating insufficient peer support. The combination of these two factors may lead to a relatively low level of health literacy in terms of information acquisition. Therefore, targeted interventions should be implemented at the primary healthcare level, such as establishing dedicated breast cancer education and awareness programs for these patients. Additionally, nurses should proactively provide personalized health education during clinical encounters, recommend easily understandable educational resources (such as videos and animations), and offer guidance on accessing reliable information platforms (e.g., official health websites and authoritative public accounts). This strategy may expand patients’ access to health information channels and enhance support for continuous learning and self-management.

Patients with moderate health literacy accounted for 29.3%. This group exhibited a moderate level of overall health literacy, with marginally higher scores in the health improvement willingness dimension of the HeLMS. Specifically, they actively monitored their health needs and were willing to invest time in enhancing their health status, which may be associated with relatively strong self-management awareness and self-care confidence (24). Within this profile, 78.8% of patients had undergone surgery, and 67.3% were at early disease stages (0–II), which may help explain helps explain their moderate health literacy pattern. For patients with early-stage disease, surgical treatment is typically the primary and preferred intervention, with the goal of improving prognosis through radical resection. For postoperative patients, targeted postoperative education can effectively improve health literacy and self-management abilities (25). For example, early standardized functional exercises help reduce complications such as ipsilateral upper extremity flap adhesion, limb edema, joint stiffness, and muscle atrophy (26), thereby improving prognosis. However, the long-term and sustained benefits of such interventions have not been fully validated. Therefore, for such patients, nurses should strengthen postoperative rehabilitation and long-term management education. This can be achieved through workshops or group discussions to help them understand complex treatment plans. By setting personalized, measurable short-term and long-term goals tailored to their rehabilitation stage, nurses can further enhance patients’ health literacy and promote the development of stable and sustainable long-term health literacy improvement programs.

Patients with high health literacy accounted for 61.5%. This group demonstrated strong health awareness and efficient information processing abilities, actively acquiring and applying health knowledge to guide health-related behaviors (27). However, within this profile, willingness to provide financial support was relatively low, primarily reflected in reluctance to cover medical expenses. This may be attributed to the substantial disease burden and pressure from family responsibilities (26). Among the study subjects, 70.2% of patients had undergone chemotherapy, and 36.0% were in stages III-IV, requiring ongoing financial support for neoadjuvant or adjuvant chemotherapy, as well as the management of side effects such as hair loss and fatigue. Evidence suggests that 51% of breast cancer survivors experience varying degrees of financial toxicity, with cancer-related financial toxicity being more prevalent among breast cancer patients in China (28). The statutory retirement age for women in China typically ranges from 55 to 60. Within this group, 80.7% of patients were under 60 years of age, and 39.5% had a monthly household income of no more than 5, 000 yuan. These patients must cover non-medical expenses, including daily living costs and their children’s education, which limits their ability to save and increases their vulnerability to financial strain. Furthermore, 23.7% of patients were under the age of 45. For this younger, working population, a cancer diagnosis and treatment may lead to reduced job stability, resulting in income fluctuations and reduced benefits, which further exacerbate the risk of financial toxicity. Therefore, nurses should emphasize the importance of standardized chemotherapy in improving quality of life and ensuring treatment adherence during patient consultations, using real-life survival stories to help foster positive treatment beliefs. They should also clearly explain medical insurance reimbursement policies, targeted assistance programs, and charitable medication donation initiatives to help alleviate the burden of financial toxicity.

### 4.2 Quality of life variations among breast cancer patients across different health literacy profiles

The results of this study indicate that there are statistically significant differences in quality of life among breast cancer patients across different health literacy profiles (all *P* < 0.05). Patients in the high health literacy group had the highest quality-of-life scores, those in the moderate health literacy group had intermediate scores, and those in the low health literacy group had the lowest scores. Previous research has shown that age, BMI, and health literacy together explain 20.0% of the total variation in quality of life among breast cancer patients, with health literacy alone being the primary predictor, accounting for 18.8% of the variance (6). Additionally, health literacy is associated with cognitive status, depression levels, activities of daily living, and physical functioning (29). As a protective factor, high health literacy can effectively improve patients’ quality of life. Patients with higher health literacy can access health information through diverse channels, enabling them to gain a thorough understanding of disease knowledge, treatment plans, and key postoperative recovery points. When communicating with nurses, they can clearly articulate their concerns and needs, effectively comprehend and adopt professional guidance, thereby enhancing their disease management processes and health outcomes. Conversely, low health literacy is considered a significant risk factor. Patients struggle to understand specialized medical knowledge, often discontinuing treatment due to misunderstanding its importance or fearing adverse effects. They have difficulty effectively managing treatment-related side effects and frequently experience body image disturbances resulting from mastectomy or changes in breast shape, which can lead to reduced quality of life. Therefore, nurses should prioritize patients with low health literacy, minimize technical jargon, and use plain, easy−to−understand language. Clinical nurses should utilize a variety of communication tools, such as videos, images, and peer support group activities, to provide targeted health education and improve patients’ understanding and application of relevant information.

### 4.3 Potential factors influencing health literacy profiles among breast cancer patients

Research findings indicate that educational attainment, primary caregiver status, and having relatives or friends employed in healthcare are significant factors influencing the health literacy profiles of breast cancer patients. Compared to individuals with primary school education or below, those with higher educational attainment exhibit higher levels of health literacy, consistent with findings from international studies (13, 15, 30). Educational attainment has a substantial impact on health literacy levels, partly due to differences in cognitive ability. Patients with higher educational attainment predominantly reside in urban settings characterized by well-developed educational and healthcare resources and higher economic levels. They are more likely to comprehend breast cancer-related knowledge, have access to diverse information channels, and utilize decision-support tools to engage in shared decision-making, thereby further improving their health literacy. Nurses should prioritize educational interventions for patients in the low health literacy group, with a focus on three key domains: disease knowledge, cognitive counseling, and attitude development. This strategy aims to enhance their understanding of breast cancer risk factors, screening approaches, treatment modalities, and post-treatment rehabilitation (31).

This study found that patients for whom the primary caregiver was a spouse exhibited higher levels of health literacy, which is consistent with the findings of Høeg et al (32). This phenomenon may be attributed to the emotional and practical support that spouses can provide to patients. In fact, informal caregivers such as spouses, family members, or friends play a vital role in the treatment and recovery process of patients with breast cancer. This may be explained by a dual coping model, wherein patients and their caregivers jointly address stressors such as cancer diagnosis and treatment (33). Therefore, nurses should prioritize integrating informal caregivers into the care system, for example, through clear communication and the provision of tailored health education materials for this group, thereby jointly improving health literacy levels in both patients and caregivers.

The diagnosis and treatment of breast cancer involve substantial amounts of complex medical information, which creates considerable challenges for patients with limited health literacy. This may lead to anxiety and confusion, impeding their active participation in medical decision−making (30). This study found that patients whose friends or family members have backgrounds in the healthcare field tend to exhibit higher levels of health literacy. This may be attributed to the fact that such relatives and friends can serve as communication “bridges,” effectively narrowing the gap between professional medical knowledge and patients’ understanding, thereby enabling patients to participate more actively in shared decision-making. Nurses should adopt flexible communication strategies, employing professional terminology when communicating with patients’ healthcare-related relatives or friends, while prioritizing plain language and everyday expressions in direct communication with patients. Communication strategies should be tailored to each patient’s individual health literacy profile, and provide emotional support when necessary. This approach may optimize the patient experience and enhance their ability to engage in informed decision-making.

## 5 Conclusion

This study used latent profile analysis to categorize breast cancer patients’ health literacy into three distinct profiles: the low health literacy group, the moderate health literacy group, and the high health literacy group. Educational attainment, primary caregiver status, and whether relatives or friends work in healthcare are significant factors influencing these health literacy profiles among breast cancer patients. Additionally, this study demonstrated differences in quality of life across these profiles, providing theoretical support for the relationship between health literacy levels and quality of life among breast cancer patients.

## 6 Limitation

This study was a cross-sectional survey conducted among patients in Guangdong Province and Guangxi Zhuang Autonomous Region, resulting in limited representativeness. Future studies should consider conducting multicenter studies with larger sample sizes to further explore the factors influencing health literacy profiles among breast cancer patients, thereby providing a basis for nurses to deliver precision nursing care.

## Data Availability

The raw data supporting the conclusions of this article will be made available by the authors, without undue reservation.
