# Correction: Latent profile analysis of health literacy among breast cancer patients and comparative analysis of quality of life disparities

**DOI:** 10.3389/fonc.2026.1869930

**Published:** 2026-05-13

**Authors:** Mengwei Jiang, Bei Wu, Yi Liu, Detian Liu, Xiaoye Chen, Ting Shu, Zijun Yuan, Linxin Xie, Hongzhen Xie

**Affiliations:** 1 Department of Health Medicine, General Hospital of Southern Theater Command, Guangzhou, Guangdong, China; 2 General Surgery, General Hospital of Southern Theater Command, Guangzhou, Guangdong, China; 3 School of Nursing, Guangdong Pharmaceutical University, Guangzhou, Guangdong, China; 4 Department of Cardiology, Nanxishan Hospital (the Second People’s Hospital) of Guangxi Zhuang Autonomous Region, Guilin, Guangxi, China; 5 School of Nursing, Southern Medical University, Guangzhou, Guangdong, China; 6 School of Medicine, Yangtze University, Jingzhou, Hubei, China

**Keywords:** breast cancer, factors, health literacy, latent profile analysis, quality of life

The **Author contributions** statement was erroneously given as:

“MJ: Data curation, Investigation, Conceptualization, Formal analysis, Writing – original draft. YL: Investigation, Data curation, Writing – review & editing, Methodology. DL: Software, Writing review & editing, Investigation, Project administration, Validation. BW: Software, Writing – review & editing, Investigation, Project administration, Validation. XC: Writing – review & editing, Supervision, Formal analysis, Investigation, Software. TS: Methodology, Investigation, Software, Writing – review & editing, Validation. ZY: Validation, Supervision, Writing – review & editing, Software, Investigation. LX: Formal analysis, Investigation, Writing – review & editing, Software. HX: Writing – review & editing, Conceptualization, Project administration, Validation, Supervision, Data curation.”

The correct **Author contributions** statement is:

“MJ: Data curation, Investigation, Conceptualization, Formal analysis, Writing – original draft. BW: Data curation, Investigation, Conceptualization, Formal analysis, Writing –original draft. YL: Investigation, Data curation, Writing –review & editing, Methodology. DL: Software, Writing –review & editing, Investigation, Project administration, Validation. XC: Writing – review & editing, Supervision, Formal analysis, Investigation, Software. TS: Methodology, Investigation, Software, Writing – review & editing, Validation. ZY: Validation, Supervision, Writing – review & editing, Software, Investigation. LX: Formal analysis, Investigation, Writing – review & editing, Software. HX: Writing – review & editing, Conceptualization, Project administration, Validation, Supervision, Data curation.”

The original version of this article has been updated.

